# Global coordination of the mutation and growth rates across the genetic and nutritional variety in *Escherichia coli*

**DOI:** 10.3389/fmicb.2022.990969

**Published:** 2022-09-20

**Authors:** Zehui Lao, Yuichiro Matsui, Shinya Ijichi, Bei-Wen Ying

**Affiliations:** School of Life and Environmental Sciences, University of Tsukuba, Ibaraki, Japan

**Keywords:** genome reduction, mutator, machine learning, support vector machine, multiple linear regression, mutation rate, growth rate

## Abstract

Fitness and mutability are the primary traits of living organisms for adaptation and evolution. However, their quantitative linkage remained largely deficient. Whether there is any general relationship between the two features and how genetic and environmental variables influence them remained unclear and were addressed here. The mutation and growth rates of an assortment of *Escherichia coli* strain collections, including the wild-type strains and the genetically disturbed strains of either reduced genomes or deletion of the genes involved in the DNA replication fidelity, were evaluated in various media. The contribution of media to the mutation and growth rates was differentiated depending on the types of genetic disturbance. Nevertheless, the negative correlation between the mutation and growth rates was observed across the genotypes and was common in all media. It indicated the comprehensive association of the correlated mutation and growth rates with the genetic and medium variation. Multiple linear regression and support vector machine successfully predicted the mutation and growth rates and the categories of genotypes and media, respectively. Taken together, the study provided a quantitative dataset linking the mutation and growth rates, genotype, and medium and presented a simple and successful example of predicting bacterial growth and mutability by data-driven approaches.

## Introduction

Growth fitness and genetic mutability are the primary traits as the driving force of adaptation and evolution (Baer et al., 2007; Basan et al., 2020). The growth rate and the mutation rate are the quantitative parameters representing the adaptiveness and evolvability of the living organisms, respectively (Elena et al., 2007; Korem Kohanim et al., 2018; Zheng et al., 2020). Theoretical studies usually reported the fitness-dependent mutation rates upon mathematical simulation (Agrawal, 2002; Shaw and Baer, 2011). Experimental evolution often found elevated mutation rates along with fitness increase (Barrick et al., 2009; Kishimoto et al., 2010; Lenski, 2017; Shibai et al., 2017), which was practical to rescue the growth from genetic engineering in laboratory (Kurokawa and Ying, 2019). The highly increased mutation rate, i.e., mutator, usually resulted in a decrease in growth rate (Funchain et al., 2000; Ishizawa et al., 2015), and the fitness increase associated with the mutation rates (Couce et al., 2017; Nishimura et al., 2017). The experimental observations and theoretical assumptions suggested that the mutation and growth rates were associated. However, the quantitative linkages of the mutation and growth rates remained largely deficient. Whether and how the genetic and environmental changes disturb the relationship between mutation and growth rates are unclear, although both growth fitness and mutability are supposed to be constrained by the genetic and environmental conditions.

The previous studies strongly suggested that genetic disturbance influenced the mutation and growth rates. The reduced genome collections were constructed with bacterial strains (Hashimoto et al., 2005; Kato and Hashimoto, 2007; Mizoguchi et al., 2008; Karcagi et al., 2016) to discover the minimal genetic information essential for living systems (Mizoguchi et al., 2007; Reuß et al., 2017; Breuer et al., 2019; Rees-Garbutt et al., 2020) and to benefit the genetic engineering for substrate production (Sharma et al., 2007; Morimoto et al., 2008) and metabolic rewiring (Lee et al., 2009). The systematic assays showed that the genome reduction caused the decreased growth rates (Karcagi et al., 2016; Kurokawa et al., 2016) accompanied by increased mutation rates despite the regular mismatch repair (MMR) system (Nishimura et al., 2017). In comparison, the genetically disturbed MMR systems significantly induced the mutation rates, often associated with reduced growth rates (Ishizawa et al., 2015). These studies investigated the relationships between the mutation and growth rates in the genome-reduced and MMR deficient strains, demonstrating that both genome reduction and MMR deficiency participated in the changes in the mutation and growth rates.

Nevertheless, whether and how the coordination of the mutation and growth rates in the genome-reduced and MMR deficient strains responded to the environmental diversity remained unclear. Variation in growth media was representative of environmental diversity. Media variation could intuitively disturb the growth rates, as experimentally demonstrated with the wild-type (Aida et al., 2022), single-gene knockout (Liu et al., 2020), genome-reduced (Kurokawa et al., 2016), and laboratory-evolved strains (Kurokawa et al., 2022). The changes in growth media also adjusted the mutation rates of the wild-type and genome-reduced strains (Nishimura et al., 2017) and those of MMR deficient strains (Ishizawa et al., 2015). These experimental findings revealed that nutritional richness affected the mutation and growth rates; resultantly, it might influence the relationship between the mutation and growth rates.

As the genetic and environmental variables contributed to the mutation and growth rates and probably interrupted their coordination, whether and how these variables and parameters related to each other are intriguing questions. In the present study, we investigated whether there’s any global pattern among the mutation and growth rates, genetic and environmental variables, and, if applicable, how they coordinated with each other. Instead of the mechanistic interpretation, we attempted to construct simple models for quantitative understanding of the genetic and environmental contributions to the coordination of the mutation and growth rates. As a pilot survey, the genome reduced strains, and the newly constructed mutator strains were assayed to examine the genetic contribution. Three media representing varied nutritional richness were tested to investigate the environmental contribution. Both theoretical regression and machine learning were applied to discover a quantitative and global linkage taking the genotype, the medium, and the mutation and growth rates into account.

## Results and discussion

### Nutritional richness mediated changes in the mutation and growth rates

**FIGURE 1:**
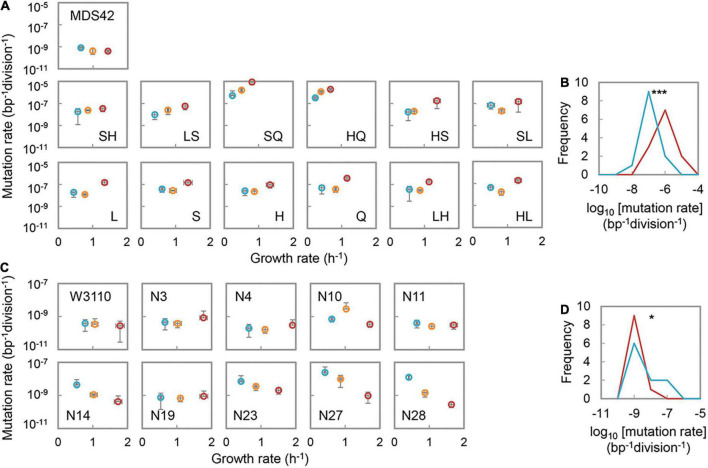
Nutritional richness-dependent mutation and growth rates. **(A)** Mutation and growth rates of the MDS collection in various media. The abbreviations of the strains, i.e., the genes deleted, are indicated. S, H, L, and Q represent the deleted genes of *mutS*, *mutH*, *mutL*, and *dnaQ*, respectively. Double letters indicate the double deletion of the genes. Blue, orange, and red circles represent the media of M63, MAA, and LB, respectively. Standard errors of both mutation and growth rates are indicated (*N* = 3∼6). **(B)** Distributions of the mutation rates of the MDS collection. Red and blue indicate the media of LB and M63, respectively. Frequency represents the number of strains. Statistical significance is indicated (***p < 0.001). **(C)** Mutation and growth rates of the KHK collection in various media. The abbreviations of the strains are indicated. Blue, orange, and red circles represent the media of M63, MAA, and LB, respectively. Standard errors of both mutation and growth rates are indicated (*N* = 3∼6). **(D)** Distributions of the mutation rates of the KHK collection. Red and blue indicate the media of LB and M63, respectively. Frequency represents the number of strains. Statistical significance is indicated (*p < 0.05).

To investigate the nutritional-dependent changes in mutation rate, two *E. coli* strain collections were analyzed. The MDS collection was newly constructed from the cleaned genome strain of MDS42 by deleting the genes participating in the mismatch repairing and DNA replication fidelity systems, i.e., *mutS*, *mutH*, *mutL*, and *dnaQ* (Echols et al., 1983; Rewinski and Marinus, 1987; Yang, 2000). A total of 13 strains were used in the present study. The KHK collection was a reduced genome library constructed from the wild-type strain of W3110, previously (Mizoguchi et al., 2008). Ten strains of varied genome sizes in the KHK collection were chosen for the test, as described previously (Nishimura et al., 2017). Repeated tests showed that an increase in mutation rates in response to the nutritional enrichment was observed in the MDS collection. However, the mutation rate of the reduced genome, i.e., their parent strain MDS42, was likely to be decreased in rich media (Figure 1A). Although the degrees of change in mutation rates were varied, the directional shift of the distributions of mutation rates from poor (M63) to rich (LB) media was highly significant (*p* = 8e-7) (Figure 1B). The tendency was consistent with the directional changes in mutation rates of the MG collection in response to the changes in media (Supplementary Figure 1), although the dataset was obtained with different assay methods previously (Ishizawa et al., 2015). Nutritional enrichment increased the mutation rates of the mutators, independent of the genomes.

On the other hand, a decrease in mutation rates of the KHK collection in response to the nutritional enrichment was identified (Figure 1C). The distribution of mutation rates was slightly but significantly (*p* = 0.03) shifted from low to a high level in response to the medium alteration from LB to M63 (Figure 1D). The nutritional richness mediated changes in mutation rates were somehow reasonable. Increased mutation rates of the wild-type genomes and the mutators more often caused the mutations that triggered the damage to the cells; however, the sufficient nutrients in the rich media could compensate for the damages caused by mutation. The specific nutrients in the medium might have buffered the fitness decrease caused by the mutations (Casanueva et al., 2012; Kinsler et al., 2020; Eisner et al., 2022), which allowed high mutability. Increased mutation rates of the reduced genomes in the poor media could be elucidated by the severe environmental stress-induced mutagenesis (Maharjan and Ferenci, 2017; Frenoy and Bonhoeffer, 2018). The differentiated directions of the changes in mutation rates responding to the nutritional changes suggested that the large deletion of genomic fragments and the interruption specifically in replication fidelity contributed to the mutability differentiation. Note that the growth rates were universally increased in the order of M63, MAA, and LB, regardless of the genotypes (Figures 1A,C), which well-reflected the expected contribution of the nutritional richness to the growth fitness.

### Genetic disturbance mediated changes in the mutation and growth rates

**FIGURE 2:**
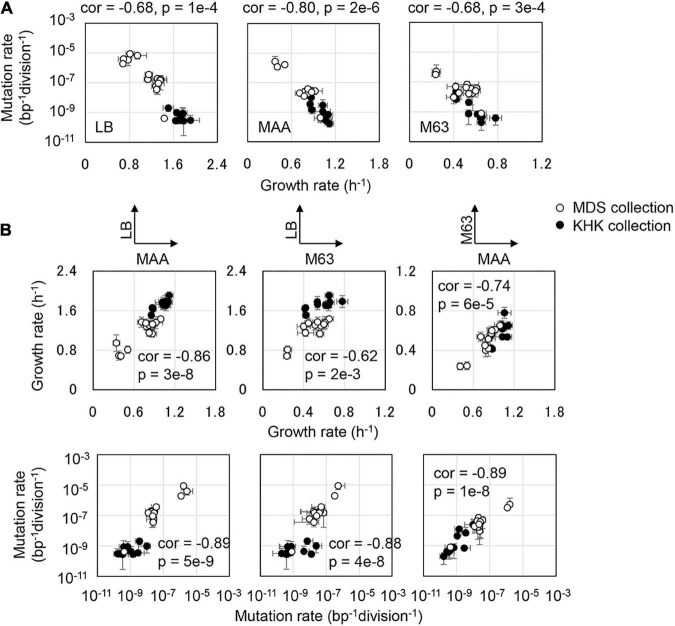
Coordinated changes in mutation and growth rates. **(A)** Correlation between growth rate and mutation rate in the defined medium. Pearson correlation coefficients, the *p* values, and the media are indicated. **(B)** Correlated changes of the mutation and growth rates in various media. The upper and bottom panels show the correlated changes in growth and mutation rates between any two different media, respectively. Open and closed circles stand for the collection of MDS and KHK collections, respectively.

A common negative correlation of growth rate to mutation rate was highly significant in all media, despite the variation in genotypes, including the reduced genomes and mutators (Figure 2A and Supplementary Figure 2). The directional changes in mutation rate were associated with the changes in growth rate. Combining the reduced genomes and the mutators resulted in the global correlation between mutation and growth rates in the three media (Figure 2A). Even if the three datasets were combined, both the trend of negative correlation between the mutation and growth rates and the differentiation in the slopes remained clearly (Supplementary Figure 2). The decreased growth rate accompanied by increased mutation rate was independent of the nutritional richness. The global parameters of the mutation and growth rates were stringently connected, as a common phenomenon evidently in reduced genomes, i.e., the MDS and KHK collections. Although the fitness effect of mutations depended on the genomic background (Wang et al., 2016), the trajectory across the various genotypes indicated the coordination of the mutability to the fitness.

A simple regression (Eq. 1) showed that all strains of various genotypes followed a common trajectory formed by the mutation and growth rates, and the magnitudes of the correlated changes were medium-dependent.


(1) μ=iLN(M/iM)∞/α

Here, M_*i*_ and μ_*i*_ represent the mutation rate and the corresponding growth rate in a defined condition. M_∞_ and α indicate the maximal mutation rate when the growth rate dropped to zero and the magnitude of the growth decrease caused by the increased mutation rate (slope), respectively. Both M_∞_ and α are nutritional dependent but genotype independent and could be calculated according to the experimental data sets. The slope of α was −9.5, −12.6, and −12.8 in LB, MAA, and M63, respectively (Supplementary Figure 2). Increased mutation rate led to a similar degree of growth decrease in MAA and M63 but a more significant reduction in LB. In addition, M_∞_ were 1e-3, 4e-4, and 7e-6 bp^–1^division^–1^ in LB, MAA, and M63, respectively, suggesting that the nutritional richness decided the maximal mutation rate. Since the genome size was approximately 4 × 10^6^ bps, the poor nutritional condition allowed only a few mutation(s) per genome compared to the rich nutritional condition that allowed more than 1,000 mutations per genome.

**FIGURE 3:**
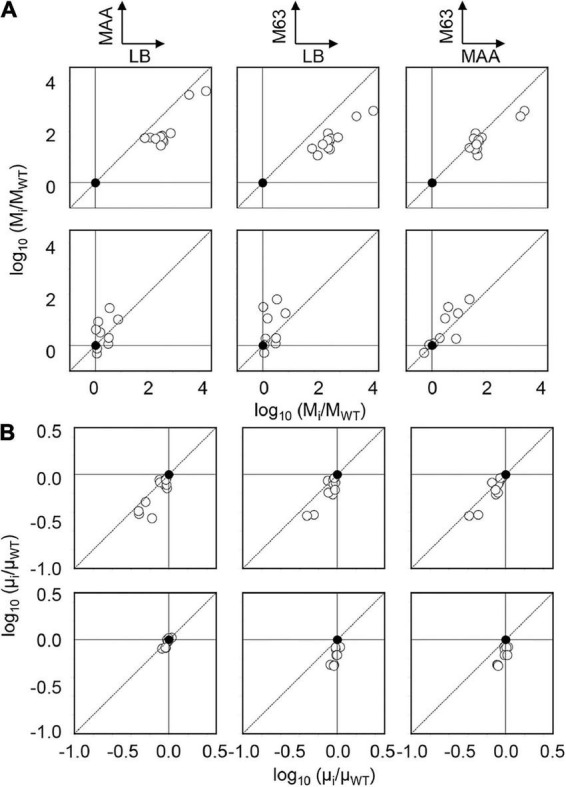
Changes in mutation and growth rates. The magnitudes of the changes in the mutation (**A**) and growth (**B**) rates due to the genetic disturbance are shown in the logarithmic scale. The upper and bottom panels indicate MDS and KHK collections, respectively. Any pairs of the three media are indicated. Closed and open circles represent the parent strain and its derivatives, respectively.

In addition, the positive correlations of growth and mutation rates between any pair of the media were observed (Figure 2B). It demonstrated that the changes in nutritional richness maintained the order of the growth fitness and mutability. However, the direction of the changes in mutation rates due to the genetic disturbance was somehow differentiated (Figure 3A). The changes in mutation rates caused by MMR deficiency were more significant in rich media (Figure 3A, Upper) compared to the changes mediated by genome reduction, which were more prominent in poor media (Figure 3A, Bottom). Nevertheless, the changes in growth rates in response to the nutritional alteration were roughly identical in both collections (Figure 3B). Taken together, it was highly intriguing that the negative correlations between the growth rate and the mutation rate were in common once the medium was determined; however, the directions of the nutritional responsivity of the mutation rate were reversed decided by the types of genetic disturbance.

### Comprehensive association of the mutation and growth rates with genotypes and media

The exhaustive tests identified a common trajectory directed the correlated changes in the mutation and growth rates across varied genotypes at all nutritional levels. The results were highly consistent with the previous studies on the correlation of the mutation and growth rates in MG1655 (Ishizawa et al., 2015), as well as the coordinated changes in the mutation and growth rates mediated by genome reduction (Nishimura et al., 2017). It suggested a trade-off relationship between the growth rate and the mutation rate in common, which was feasible in evolution (Nishimura et al., 2017; Kang et al., 2019). Experimental evolution of the KHK collection demonstrated that equivalent generation led to equal numbers of genome mutations fixed on the reduced genomes but none in the wild-type genome; nevertheless, their growth rates were improved comparably (Kurokawa et al., 2022). To further confirm the generality of the relationship between the mutation and growth rates, the evolved KHK collection strains were additionally tested. The increased growth rates resulting from the experimental evolution were associated with reduced mutation rates (Supplementary Figure 3). However, the mutations fixed in experimental evolution were unrelated to the DNA replication fidelity and MMR system (Kurokawa et al., 2022). The correlated changes in the mutation and growth rates were in common. The trade-off mechanisms were crucial in bacterial competition and coexistence (Ferenci, 2016) and shaped the diversity of species in eco-evolution (Farahpour et al., 2018). The negative correlation of the mutation and growth rates across the genetic variation well-agreed with those reported trade-offs, which might be relied on the cost-benefit working principle in living systems (Eames and Kortemme, 2012; Weisse et al., 2015; Erickson et al., 2017).

**FIGURE 4:**
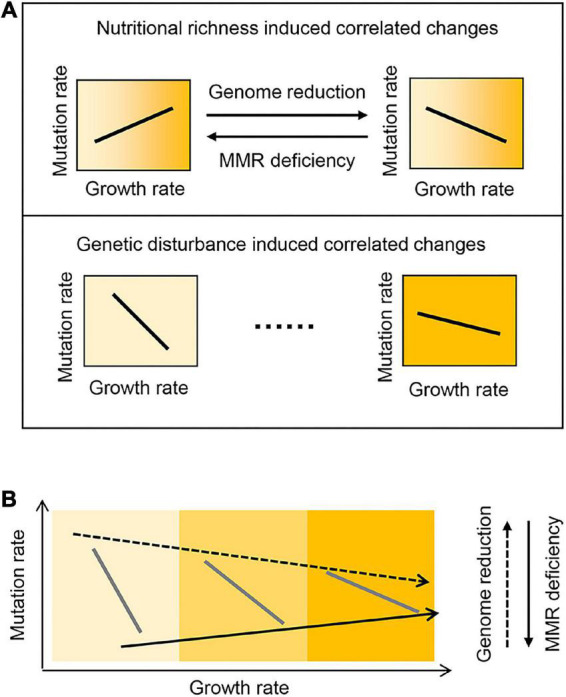
Schematic drawing of the relationships among the mutation and growth rates, medium, and genotype. **(A)** Experimentally observed correlations of the mutation and growth rates. **(B)** Hypothesized universal coordination of the mutation and growth rates across the genetic and nutritional varieties. Gradation in yellow represents the variation in nutritional richness.

Intriguingly, genome reduction and MMR deficiency showed differentiated directions of the correlated changes in the mutation and growth rates in response to nutritional variety (Figure 4A, Upper). The positive correlation of the mutation rate to the growth rate triggered by MMR deficiency supported the theoretical framework, which proposed that the mutation accumulation rate increased with the cell division rate across species (Gao et al., 2016). In contrast, both genetic disturbances presented the universal direction in defined media (Figure 4A, Bottom). The present study first observed the reversibility in the direction of the medium-dependent changes in mutation rate. The compensability between genome reduction and MMR deficiency might be tuning the DNA replication errors regulated by the gene expression to maintain the balance between fitness and evolvability in response to environmental changes. Suppose genome reduction and MMR deficiency had caused the correlated changes of the mutation and growth rates in an identical but not reverse direction. In that case, it must have been disadvantageous for survival, as the high mutation rates might restrict the adaptative evolution for growth improvement (Sprouffske et al., 2018). Theoretically, the maximal mutation rate was assumed to be 10^–5^∼10^–3^/bp/division in different growth media (Eq. 1, Figure 2), which were higher than those of RNA viruses (Krašovec et al., 2017; Duffy, 2018). However, the experimentally acquired mutation rates were lower than ∼10^–6^/bp/division, independent of the genetic backgrounds of *E. coli* (Ishizawa et al., 2015; Krašovec et al., 2017; Shibai et al., 2017; Ramiro et al., 2020). As the genome size of *E. coli* was ∼4 Mb, the mutation rate detrimental to survival was assumed to be ∼10^–6^/bp/division, which led to roughly one division for one mutation.

The comprehensive association of the mutation and growth rates with the genotype (genetic interruption) and medium (nutritional richness) was supposed to be universal (Figure 4B). The biological mechanism in charge of their connections, if it existed, was largely unknown. It was somehow hard to be addressed so far. A preliminary analysis showed that the expression levels of the genes participating in the DNA replication and mismatch repair were either increased or decreased in the KHK collection strains (Supplementary Figure 4). However, these reduced genomes maintained the regular DNA replication and mismatch repair systems (Mizoguchi et al., 2008; Kurokawa et al., 2016). It indicated that the biological mechanisms could not directly explain the correlated changes in mutation rates. High-throughput transcriptome analysis was required to acquire the big data linking the global changes in gene expression to the mutation and growth rates. Instead, the theoretical understanding of the relationships might be practical. Suppose the relationships between the mutation and growth rates, the medium, and the genotype were universal. Any of them might be theoretically estimated according to the other three parameters. The prediction with the regression and machine learning approaches was challenged to achieve a quantitative understanding of the cooperative relationships among the four parameters.

### Estimation of the mutation and growth rates by multivariable regression

**FIGURE 5:**
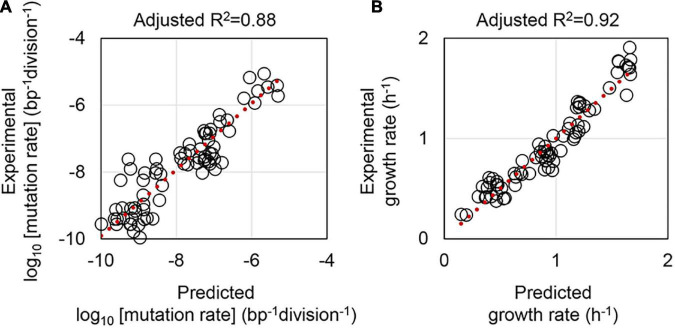
Prediction of the mutation and growth rates by multiple linear regression. Adjusted R2 denotes the accuracy of multiple linear regression. Red dotted lines indicate the slope of 1. **(A,B)** Represent the mutation and growth rates, respectively. The corresponding statistics are summarized in Supplementary Table 3.

Whether the mutation and growth rates could be estimated according to the other parameters was tested. Multiple linear regression (MLR) was applied, where the logarithmic values of mutation rates were used, and both the media and the genotypes were set as the numerals (Supplementary Table 2). Note that the genotype, which represented the combination of the reduced genome and mutator genotype, was evaluated in two different modes in MLR. Whether the genome reduction and the MMR deficiency interacted or not were defined as interactive and additive modes, respectively. MLR showed that the mutation rate could be well-estimated according to the growth rate, the medium, and the genotype (Figure 5A), independent of the mode applied for the genotype (Supplementary Table 3). The growth rate was also successfully predicted by MLR according to the mutation rate, the medium, and the genotype in the mode of interactive (Figure 5B). Although the prediction accuracy of the additive mode remained equivalent to that of the interactive mode, the contribution of the genotype to the regression (prediction) was insignificant (Supplementary Table 3). The results suggested genome reduction and deficient MMR.

Moreover, another dataset was employed to address whether the predictivity of the mutation and growth rates was general, as varied strains and methods might cause different consequences. The *E. coli* strain collection derived from MG1655 was adopted from the previous study (Ishizawa et al., 2015). MLR of the three different datasets comprising the MDS, KHK, and MG collections, that is, the W3110, MDS42, and MG1655 derivatives, showed that the prediction of the mutation rate was statistically significant. However, the accuracy was much lower (Supplementary Table 4). On the other hand, the growth rate prediction was somehow insignificant (Supplementary Table 4), probably because of the differentiation in the methods of growth assay. In conclusion, the mutation and growth rates were roughly predictable to each other when the nutritional richness and genotype were decided.

### Classification of the medium and genotype by machine learning

**FIGURE 6:**
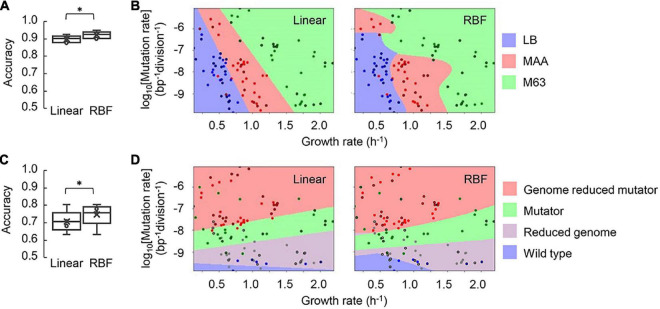
Prediction of the medium and genotype by support vector machine. **(A)** Boxplots of the evaluation metrics of the machine learning models for medium classification. The accuracy of predicting the growth medium with either linear or RBF model of SVM is shown. Five independent tests with the data unused for training are indicated. Statistical significance is indicated (*p < 0.05). **(B)** Visualization of the medium clusters. One of five tests is shown as an example. Color variation represents the medium variety as indicated. The data points are signified by the dots, in which those for the test are circled by black lines and the rest for the training. **(C)** Box plots the evaluation metrics of the machine learning models for classifying the genotype. The accuracy of predicting the growth medium with either linear or RBF model of SVM is shown. Five independent tests with the data unused for training are indicated. **(D)** Visualization of the genotype clusters. One of five tests is shown as an example. Color variation represents the genotype variety as indicated. The data points are signified by the dots, in which those for the test are circled by black lines and the rest for the training.

Alternatively, whether the medium and the genotype could be clustered according to the mutation and growth rates were investigated by machine learning (Jordan and Mitchell, 2015), which was beneficial for discovering the dataset with unclear mechanisms relating to metabolism (Cuperlovic-Culf, 2018; Kim et al., 2020), genetics (Libbrecht and Noble, 2015; Schrider and Kern, 2018), evolution (Wang et al., 2018) and population dynamics (Ashino et al., 2019; Cao et al., 2020; Gilpin et al., 2020). Here, the support vector machine (SVM) was applied to classify the three media (i.e., LB, MAA, and M63) and the four genotypes (i.e., reduced genome, mutator, genome reduced mutator, and wild type). All three datasets (i.e., the MDS, KHK, and MG collections) were subjected to SVM machine learning. The linear regression and the radial basis function (RBF) models were tested, as both the quantitative parameters of the mutation and growth rates and the qualitative parameters of the genotype and medium were comprised in the datasets. The growth medium was well-predicted from the mutation and growth rates by both models (Figure 6A). The RBF model presented a higher accuracy of medium classification, either in testing (Figure 6A) or in training (Supplementary Figure 5). Visualization of the medium variety clearly showed the discontinuous edges/areas of MAA in the landscape formed by the mutation and growth rates in the RBF model (Figure 6B). The unique landscape in the MAA medium might be attributed to either nutritional or generic biases. The MAA medium was rich in amino acids and remained poor in other nutritional elements, such as glucose. As fast growth preferred catabolism of amino acids and reduced glucose uptake in *E. coli* (Zampieri et al., 2019), the enriched amino acids in MAA possibly benefited the growth even with a high mutation rate. In addition, the reduced genomes had low redundancy in non-essential sequences. As the genomic sequences were related to the turnovers of carbon, nitrogen, and sulfur (Baker et al., 2015), genome reduction might disturb the cycling of these essential elements, causing slow growth even with a low mutation rate. Taken together, the coordination of amino acids and glucose catabolism and the reduced redundancy of the genome might trigger the discontinued landscape of the mutation and growth rates.

In addition, the two SVM models predicted the genotype with comparable accuracy in the training (Supplementary Figure 5). Nevertheless, the prediction accuracy of the RBF model was significantly higher than that of the linear model in the test (Figure 6C). Both models could roughly categorize the four genotypes (Figure 6D). It demonstrated that a simple SVM machine learning model could provide an acceptable classification of the genotype, despite the prediction accuracy being lower than that of classifying the medium. An increased number of data points were required to achieve better precision.

In summary, the present study found an intriguing global correlation of the growth rate to the mutation rate across a wide genetic variety and nutritional variation. As the mutation rate was proposed as a plastic trait associated with population density across domains of life (Krašovec et al., 2017), the cooperative relationship between the mutation and growth rates was supposed to be universal in living systems (Shaw and Baer, 2011). The results expanded the previous findings of the correlated changes in the mutation and growth rates with the defined genotypes (Wang et al., 2018) or the defined conditions (Ishizawa et al., 2015; Nishimura et al., 2017). It indicated the fundamental working principle in maintaining cellular homeostasis. Genetic disturbance in the genome length or the MMR system led to the coordinated changes of mutation rate and growth fitness in common but the reverse directional changes in response to nutritional richness. It strongly suggested that the genetic deficiency could partially compensate for each other, that is, the genome reduction might recover the damage caused by the disturbed MMR system to some extent. The simple regression and machine learning models connected the growth rate, mutation rate, genotype, and medium well. The successful connection among these parameters indicated that the fitness and evolvability were intrinsically associated in living cells, regardless of the genetic, and environmental interruptions. To develop an advanced ML model taking the underground biological mechanisms into account, the transcriptome analysis is required to connect the mutation and growth rates to the gene expression. The ML predicted mechanisms remain to be challenged in the future.

## Materials and methods

### Genetic construction of the MDS mutators

Genome-reduced mutators were constructed by deleting the genes that participated in mismatch repair or proofreading, i.e., *mutH*, *mutS*, *mutL*, and *dnaD*, from the reduced genome MDS42 (Pósfai et al., 2006). Genetic deletion of the single genes and any pairs of these genes was performed as described previously (Ying et al., 2014; Ishizawa et al., 2015). Briefly, the deletions were induced *via* standardized λ-red homologous recombination (Kirill and Barry, 2000; Ying et al., 2010). The chloramphenicol resistance gene was used repeatedly as a selection marker in each deletion. Genetic and phenotypic verification of the transformants was carried out as previously described (Ying et al., 2014; Ishizawa et al., 2015). The primers for genetic deletion and PCR confirmation were reported previously (Ishizawa et al., 2015). A total of 13 mutator strains were successfully constructed. One out of 13 mutators failed to grow in the minimal medium, as the genetic construction was performed with the rich medium. Note that the gene circuit sequence was previously used in various studies (Ying et al., 2014, 2015, 2017; Ishizawa et al., 2015; Kishimoto et al., 2015; Shibai et al., 2017; Lu et al., 2022), and no experimental bias was observed.

### Media

Three different media of LB, MAA, and M63 were used for cell culture, representing rich, supplementary, and poor nutritional conditions. The LB medium was commercially available (Luria-Bertani, Sigma). The M63 and MAA media were the minimal medium and the minimal medium supplied with 20 amino acids, respectively, prepared as described previously (Kurokawa et al., 2016; Nishimura et al., 2017).

### Growth assay

The growth dynamics in the three media were assayed as described previously (Kurokawa et al., 2016; Kurokawa and Ying, 2017). The cell culture was diluted and loaded to the 96-well plate (Costar), which was incubated in a plate reader (Epoch2, BioTek) with a rotation rate of 600 rpm at 37°C. The cell growth was detected at an absorbance of 595 nm, reading at an interval of 30 min or 1 h for 24 to 48 h. The growth curves were obtained for each well. Repeated tests (*N* = 6∼12) were performed to acquire the growth curves in each condition. The growth rates were calculated as described previously (Kurokawa et al., 2016; Nishimura et al., 2017).

### Fluctuation test

The mutation rate was estimated by the fluctuation test according to resistance to the antibiotic nalidixic acid, as described previously (Ishizawa et al., 2015; Nishimura et al., 2017). In brief, the number of cells was counted using a CFU assay. The *E. coli* cell cultures in the exponential phase were diluted and plated onto ∼10°LB plates for CFU assay. Only the number of colonies per plate ranging from 10 to 500 was considered reliable for calculating the CFU. Approximately 30 tubes of identical cell culture were used to evaluate the frequency of mutagenesis for each test. At least three repeated tests were performed for each strain at each medium, and more than 8,000 agar plates were used. The mutation rates were calculated as described previously (Kishimoto et al., 2010; Ishizawa et al., 2015). Note that the mutation rate was evaluated based on the emerging frequency of nalidixic acid resistance; nevertheless, we previously verified that the relative mutation rates did not change in response to different antibiotics (Ishizawa et al., 2015).

### Data acquisition

The mutation and growth rates of the MDS collection (16 strains) were obtained in the present study. The reduced genomes of *E. coli* strains, i.e., the derivatives of W3110, were randomly selected from the KHK (Kyowa Hakko Kirin) collection (Mizoguchi et al., 2008), as described previously (Nishimura et al., 2017). Note that the KHK collection strains were constructed in an accumulative deleted manner. The mutation and growth rates of the ten KHK strains were partially adopted from our previous report (Nishimura et al., 2017). Additionally, the mutation and growth rates of the MG collection, which included the wild-type genome MG1655 and the nine derivative mutators, were acquired from the previous study (Ishizawa et al., 2015). The data details of the three collections are summarized in Supplementary Table 2. The RNA sequencing dataset of the KHK collection grown in the M63 medium was acquired from the DNA Data Bank of Japan (DDBJ) under the accession number DRA13430. Global normalization of the read counts (raw data) was performed as described previously (Ying and Yama, 2018). The relative expression levels of the genes participating in the DNA replication fidelity was analyzed (Supplementary Table 5).

### Multiple linear regression

Multiple linear regression (MLR) was performed with Python, as described previously (Aida et al., 2022). The media of LB, MAA, and M63 were represented as 1, 0, and −1, respectively. The *E. coli* strains of the reduced genome or MMR deficiency (mutator) were commonly indicated as one unit, and those of wild-type genome or non-mutator were set to zero. Genome reduction and MMR deficiency were categorized in the genotype, which was calculated by multiplying or adding the values of 1 or 0. Whether their relationship was considered interactive or additive, the two values representing genome reduction and MMR deficiency were multiplied or added, respectively. The logarithmic values of mutation rates were used in the MLR analysis. A total of four global parameters, i.e., growth rate, mutation rate, genotype, and medium, were finally subjected to the analysis. Ordinary least squares (OLS) regressions of the mutation and growth rates were performed with the three parameters of medium, genotype, and mutation or growth rate, respectively. The package of “stats” in Python was used, and the parameter estimation method of “ols” was applied.

### Machine learning with support vector machine

Support vector machine (SVM) was performed with Python and using the “svm” module in the package of scikit-learn, as previously reported (Aida et al., 2022). Briefly, the data points were randomly divided into two sets for the training and testing in ML as commonly performed (Zhou, 2021). The “random” function in the “svm” module was used to divide the whole dataset into the training and test datasets in the 60 to 40%. Five repeated training and test were conducted to evaluate the reliability of the SVM models. The linear and the radial basis function (RBF) methods were tested. Five-fold nested cross-validation searching the hyperparameters of C and gamma from 0.001 to 100 in increments of 10-fold was performed. The other hyperparameters were all used as default. Finally, in the “linear” method, C was set to 10 and 100 for classifying/predicting the medium and the genotype, respectively. In the “RBF” method, C and gamma were set to 10 and 1 and 100 and 0.01 for categorizing/predicting the medium and the genotype, respectively. The score of fivefold nested cross-validation was calculated by macro-averaging. The accuracy of the models was estimated according to the confusion matrix.

## Data availability statement

The original contributions presented in this study are included in the article/Supplementary material, further inquiries can be directed to the corresponding author.

## Author contributions

B-WY conceived the research. ZL, YM, and B-WY analyzed the data. SI, YM, and B-WY performed the experiments. B-WY and ZL wrote the manuscript. All authors have read and approved the final manuscript.
