# Supplementary material for: Global coordination of the mutation and growth rates across the genetic and nutritional variety in Escherichia coli
Source: Front Microbiol. 2022 Sep 20;13:990969. doi: 10.3389/fmicb.2022.990969 (PMC9530902; doi:10.3389/fmicb.2022.990969)
Supplement: Supplementary file 1 [file Presentation_1.PDF]

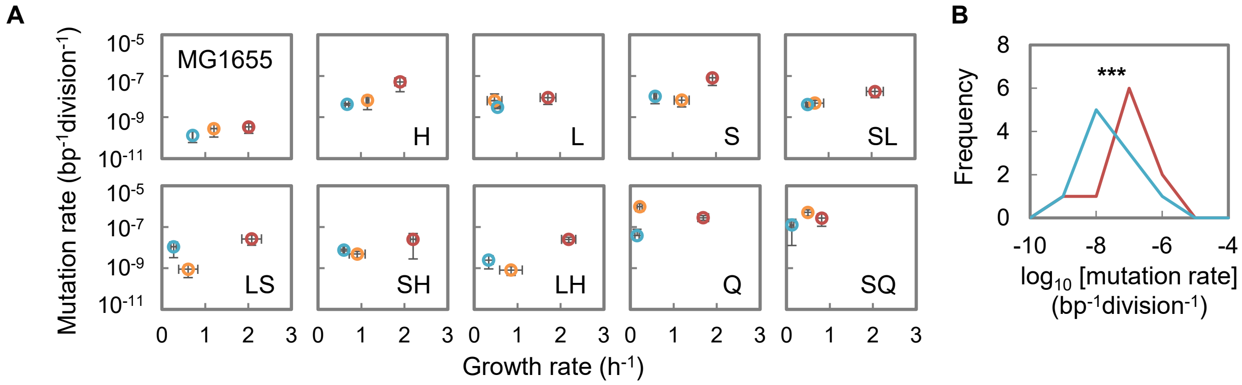

Figure S1 Nutritional richness-dependent mutation and growth rates of the MG collection. A. Mutation and growth rates in various media. The abbreviations of the strains, i.e., the genes deleted, are indicated. S, H, L and Q represent the deleted genes of *mutS*, *mutH*, *mutL* and *dnaQ*, respectively. Double letters indicate the double deletion of the genes. Blue, orange and red circles represent the media of M63, MAA and LB, respectively. Standard errors of both mutation and growth rates are indicated (N=3~6). **B.** Distributions of the mutation rates. Red and blue indicate the media of LB and M63, respectively. Frequency represents the number of strains. Statistical significance is indicated (***, $p < 0.001$).

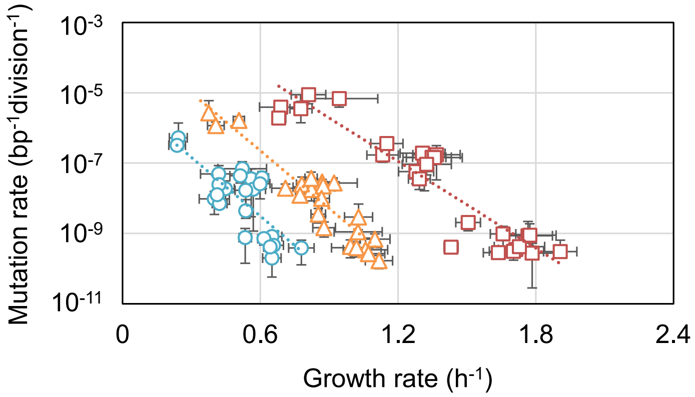

Figure S2 Correlations of the mutation and growth rates in various media. Both the KHK and MDS collections are shown without differentiation. Blue circles, orange triangles and red squares represent the media of M63, MAA and LB, respectively. Broken lines represent the logarithmic regression to the growth and mutation rates in the corresponding medium. Standard errors of both mutation and growth rates are indicated (N=3~6).

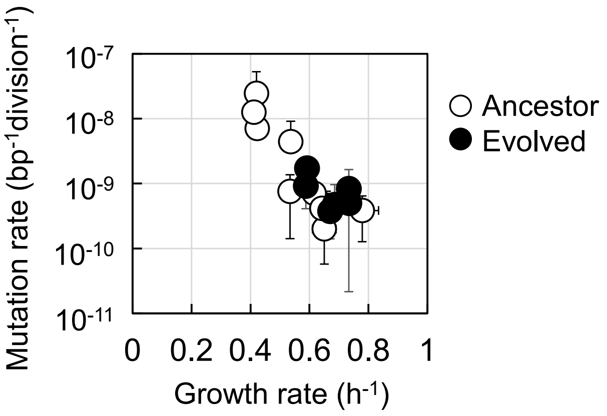

Figure S3 Coordinated changes in the mutation and growth rates. The ancestor and evolved KHK collection strains are shown in the open and closed circles, respectively. As the experimental evolution was performed in the M63 medium, the mutation and growth rates were evaluated in M63. Standard errors of the mutation and growth rates are indicated (N=3~6).

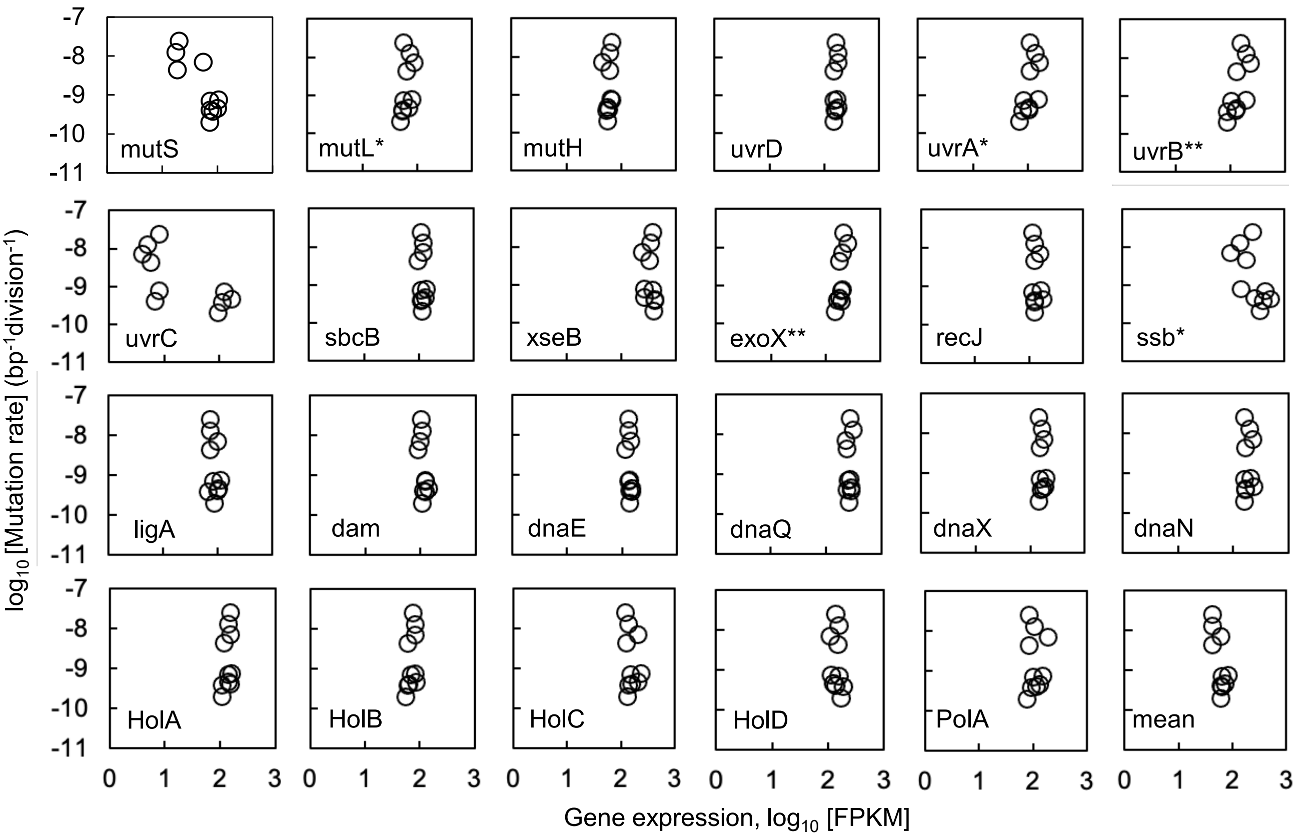

Figure S4 Relation between mutation rate and gene expression. The expression levels of the genes participating in the DNA replication and mismatch repair are shown. Both the expression levels and the mutation rates are shown in the logarithmic scale. Asterisks indicate the statistical significance of the Spearman correlation coefficients, i.e., * and ** indicate $p < 0.05$ and 0.01, respectively.

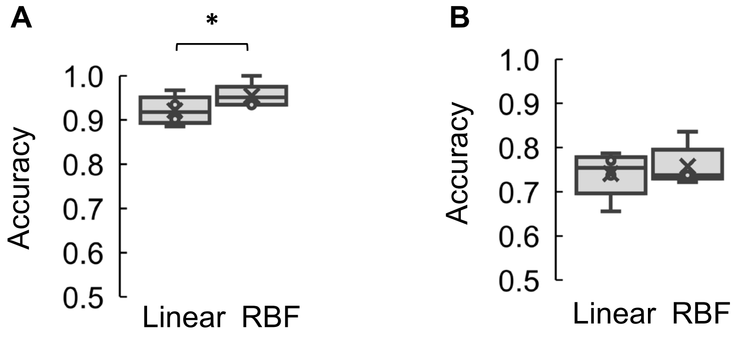

Figure S5 Boxplots of the evaluation metrics of SVM in training. The accuracy of the linear and RBF models was evaluated according to the confusion matrix. The left and right panels show the classification of medium and genotype, respectively. Five independent tests with the training data are indicated. Statistical significance is indicated (**, $p < 0.01$).
